# Essential oils from Leptospermums of the Sunshine Coast and Northern Rivers Regions

**DOI:** 10.1186/1752-153X-6-38

**Published:** 2012-05-06

**Authors:** Sarah Alison Michelle Windsor, Peter Brooks

**Affiliations:** 1 School of Science, Education and Engineering, University of the Sunshine Coast, Maroochydore DC Qld 4558, Australia

**Keywords:** Leptospermum, Laevigatum, Liversidgei, Polygalifolium, Semibaccatum, Trinervium, Whitei, Essential oils, GC-MS

## Abstract

**Background:**

Around the turn of this century, the oil yield and chemical composition of Australian Leptospermum species was analysed. Since that time, research has been focused on their use as phytomedicines. The oil yield and composition of essential oils from Australian Leptospermum species directly impacts their commercialisation for medicinal use.

**Results:**

The essential oils from Leptospermum (L.) juniperinum, L. laevigatum, L. liversidgei, L. polygalifolium, L. semibaccatum, L. speciosum, L. trinervium and L. whitei have been examined from specimens in the Sunshine Coast (Queensland) and Northern Rivers (New South Wales) Regions. Both chemotypes of L. liversidgei were observed. However, only chemotype II of L. semibaccatum and chemotype I of L. trinervium were identified. The only subspecies observed of L. polygalifolium was L. polygalifolium wallum.

**Conclusions:**

L. liversidgei chemotypes I and II have the potential for phytomedical use as antibacterial or anti-inflammatory agents. Chemotype I has the potential for use as an insect repellent and chemotype II may provide antifungal activity.

## Background

Between 1998 and 2000 Brophy and co-workers published a series of seven papers on the oil yield and chemical composition of Australian Leptospermum species. The three principal components in Leptospermum (L.) juniperinum essential oils (yield 0.1–0.2%) collected in South East Queensland (SEQ) and the Southern Highlands New South Wales (NSW) were α-pinene, 1,8-cineole and caryophyllene E, but with significant variation in the proportions of these components [1]. The principal components in L. laevigatum essential oils (yield 0.1%) from the Mid-North Coast NSW were caryophyllene E or (E,E)-farnesol [2]. L. liversidgei essential oil samples from SEQ, Northern Rivers Region NSW and Yarra Valley and Dandenong Ranges Region Victoria showed a range of oil yield from 0.3–2.7% and identified two extremes in chemical composition (high in isopulegol and citronellal and high in neral and geranial) [3]. L. polygalifolium wallum essential oil samples from SEQ showed a range of oil yield from 0.2–0.8% and was composed primarily of the monoterpenes: α-pinene, β-pinene and 1,8-cineole; but the sesquiterpene alcohols did not contribute much to this oil [4]. Monoterpenes (α-pinene and β-pinene) predominated in L. semibaccatum chemotype II essential oils (yield 0.04–0.2%) collected in SEQ, but the sesquiterpenes: caryophyllene E, bicyclogermacrene and δ-cadinene; and the alcohols: globulol, spathulenol, cadinol and muurolol; were also present in significant amounts [2]. The principal component of L. speciosum essential oils (yield 0.5–1.3%) collected from SEQ and Northern Rivers Region NSW was α-pinene [5]. L. trinervium chemotype I essential oil samples from SEQ and the Southern Highlands NSW showed a range of oil yield from 0.1–0.4% and contained significant quantities of α-pinene, sabinene, 1,8-cineole, p-cymene, p-cymen-8-ol, caryophyllene E, viridiflorene, germacrene D, bicycolgermacrene, globulol, viridiflorol and spathulenol [2]. The main monoterpene hydrocarbon, sesquiterpene hydrocarbon and oxygenated terpene found in L. whitei essential oils (yield 0.2–0.3%) collected in SEQ were α-pinene, caryophyllene E and spathulenol, respectively [5]. Intra species variation has been proposed to be dependent on location or collecting season or both [6]. Locational and seasonal variation of essential oils from individual species have been examined in this work. The widespread occurrence of multiple antibiotic-resistant organisms in hospital and community settings suggests new antimicrobial agents, preferable with novel mechanisms of action, are required and it seemed prudent to re-examine previously superseded products such as phytomedicines [7]. As opposed to most antimicrobial agents currently used for air disinfection, essential oils are low in toxicity and could be used in different environments, while people are present. They possess high volatility that is not seen in other non-toxic antimicrobial agents. Essential oils are complex mixtures which often are superior for reducing bacterial viable counts than a single active compound in both the absolute effect and in the speed of action. This is probably caused by the presence of minor compounds, like p-cymene, which can cause swelling of the bacterial cytoplasmic membrane and makes it more permeable for active compounds [8]. Essential oils have been found to provide high zones of inhibition against methicillin-resistant Staphylococcus aureus in disc diffusion assays. Lemon grass single essential oil and Respiratory Congestion (R.C.) blended essential oil provided zones of inhibition of diameters greater than 83mm, the former containing (geranial: 39–46%, neral: 29–35%, geranyl acetate: 2–7% and geraniol: 52%) and the latter composed of (1,8 cineole: 30–36%, α-pinene: 22–29% and citronellal: 4–6%). The geranial/neral single essential oils of lemon myrtle (52/39)% and melissa (29–33/21–22)% provided 65mm and 60mm diameter zones of inhibition [9]. 1,8 cineole participates in synergistic actions to exhibit antimicrobial and anti-nociceptive properties. While 1,8 cineole exhibits little microbial activity inherently, it has been shown to enhance the lethal action of terpinene. It is hypothesised that 1,8-cineole helps permeabilise bacterial membranes, allowing the more active terpinene to enter and kill the bacterial cell [9]. The anti-nociceptive properties of 1,8-cineole, morphine and naloxone were tested on rodents using the tail-flick and hot-plate tests to reflect the spinal and supra-spinal sensitivity to painful stimuli. 1,8-cineole showed an anti-nociceptive activity compared to morphine, but not compared to naloxone, thus suggesting there is significant synergism between 1,8-cineole and morphine [10].

Essential oils have been found to possess anti-inflammatory properties. Garcinia brasiliensis (similar components to L. semibaccatum chemotype II) showed a 3 h inhibition of the inflammatory process using the rat-paw oedema model induced by carrageen administration [10]. Nitric oxide is recognised as a mediator and regulator in pathological reactions, especially in acute inflammatory responses. Cinnamomum insularimontanum (similar components to L. liversidgei chemotype I) revealed a significant inhibition of nitric oxide production (50% effective concentration 18.68μg/mL) in a nitric oxide inhibitory activity assay. Since this cell assay demonstrated anti-inflammatory activity, in vivo anti-inflammatory activity assays were also performed. Cinnamomum insularimontanum reduced the croton oil-induced oedema response in mice ears by 38% for 100μg and by 77% for 500μg per ear [11]. Macrophages take part in the innate immune responses and can also be effector cells, contributing to the resolution of these responses, such as inflammation. These cells are also able to produce a variety of cytokines. Macrophages of normal mice were pooled and incubated with lemongrass essential oil (similar components to L. liversidgei chemotype II) at 37°C for 24 h. Supernatants of cell cultures were used for cytokine determination. Cytokine production was measured by enzymelinked immunosorbent assay. Interleukin - 1β and interleukin - 6 cytokine production was inhibited at a lemongrass essential oil concentration of 50μg/mL [12].

Essential oil component vapours provided antifungal activity against Phytophthora cactorum over a broad range of air concentrations. The inhibition rates against Phytophthora cactorum of citronellol, neral, geraniol and geranial were: 100% at 28 × 10^−3^mg/mL air concentration; 38%, 41%, 50% and 51%, respectively, at 14 × 10^−3^mg/mL air concentration; 26%, 29%, 39% and 40%, respectively, at 7 × 10^−3^ mg/mL air concentration; and 0%, 22%, 35% and 39%, respectively, at 3.5 × 10^−3^mg/mL air concentration. High concentrations of essential oil component vapours were required to provide antifungal activity against Cryponectria parasitica. The inhibition rates against Cryponectria parasitica of citronellol, neral, geraniol and geranial were: 36%, 62%, 44% and 69%, respectively, at 28 × 10^−3^mg/mL air concentration; and 33%, 0%, 37% and 32%, respectively at 14 × 10^−3^mg/mL air concentration. Only citronellol and neral vapours showed weak antifungal activity, 38% and 32%, respectively, against Fusarium circinatum at the highest air concentration tested [13].

Widely used synthetic insect repellents (e.g. dimethylbenzamide) have a high insect repellent effect, but some severe adverse effects (e.g. urticarial syndrome). A substitution repellent which has a low level of toxicity to human and animals and high repellent against insects needs to be found. Citronellal is a possible substitution repellent as 30% citronellal extract provided 78% repellency against mosquitoes in an in vitro test involving humans wearing a band type instrument for releasing aroma repellent [14].

## Results and discussion

The percentage oil yield of L. laevigatum observed in this study (0.01–0.06%) was lower than found by Brophy and co-workers (0.1%) [2]. The range of percentage oil yield of L. polygalifolium wallum observed in this study (0.03–0.5%) was in the lower end of the range previously reported (0.2–0.8%) [4]. Both the L. liversidgei chemotypes examined in this study had percentage oil yields (I - 0.5–1% and II - 0.5%) consistent with the non-chemotype specific L. liversidgei range of oil yield found by Brophy and co-workers (0.3–2.7%) [3]. The percentage oil yields of L. semibaccatum chemotype II, L. speciosum and L. trinervium chemotype I observed in this study (0.06–0.2%, 0.6% and 0.2–0.4%, respectively) are consistent with the ranges previously reported (0.04–0.2% [2], 0.5–1.3% [5] and 0.1–0.4% [2], respectively). The range of percentage oil yield of L. whitei in this study (0.2–0.4%) was in the higher end of the range found by Brophy and co-workers (0.2–0.3%) [5]. The percentage oil yield of L. juniperinum observed in this study (0.3%) was higher than found by Brophy and co-workers (0.1–0.2%) [1].

**Figure 1:**
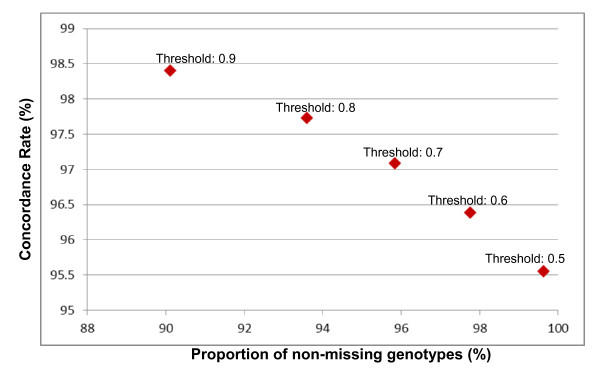
Locational variation of percentage oil yield.

**Figure 2:**
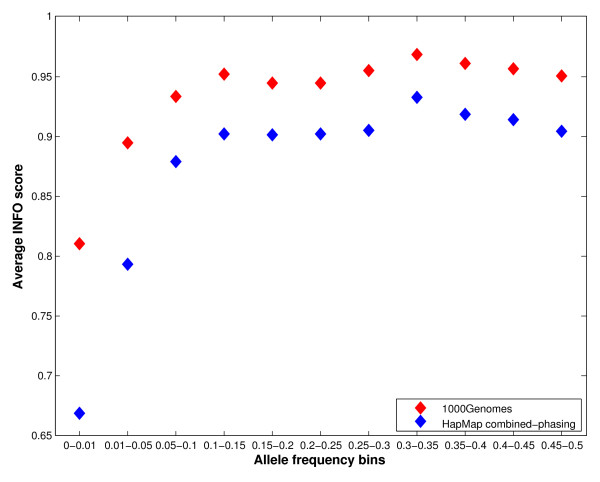
Locational variation of Leptospermum polygalifolium wallum composition.

**Figure 3:**
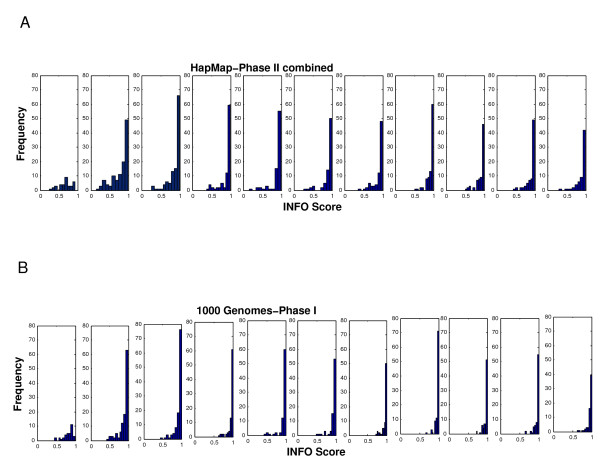
Locational variation of Leptospermum whitei composition.

Essential oils from the summer green foliage of 11 trees was used in the analysis of the locational variation of percentage oil yield and percentage composition of L. polygalifolium wallum and L. whitei. One individual tree was sampled for the results displayed in Figures 1, 2, 3 for L. polygalifolium wallum at Lennox Heads and Broadwater and for L. whitei at the University of the Sunshine Coast (USC), Lennox Heads and Broadwater. The average of the percentage oil yield and percentage composition of essential oils from two individual trees is represented in Figures 1, 2, 3 for L. polygalifolium wallum at USC and Tyagarah and for L. whitei at Tyagarah. Quantitative locational variations are given as (Average ± Standard Deviation) percentage oil yield and percentage composition.

**Figure 4:**
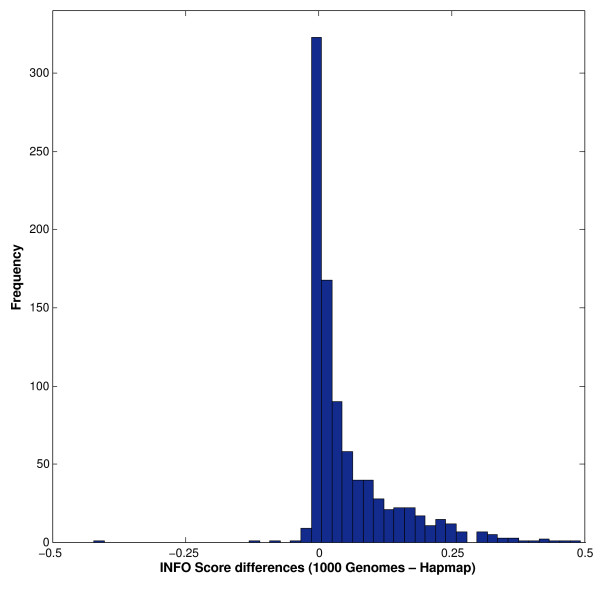
Seasonal variation of percentage oil yield.

**Figure 5:**
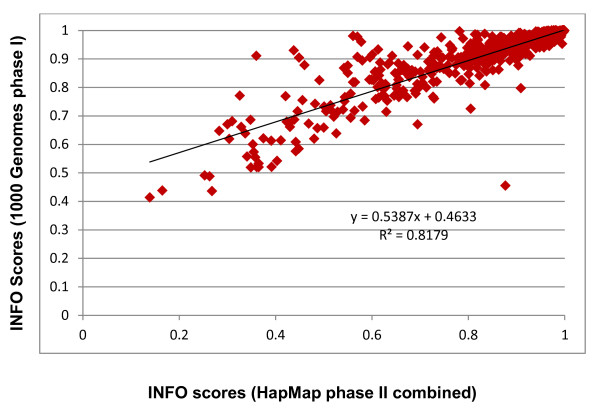
Seasonal variation of α-pinene percentage composition.

In Figure 1 the percentage oil yield of samples taken from L. polygalifolium wallum and L. whitei at different locations during summer are displayed. L. polygalifolium wallum showed significant locational variation (0.19 ± 0.17)%, whereas L. whitei showed negligible locational variation (0.35 ± 0.04)%. Figure 2 shows the locational variation of the three major constituents of L. polygalifolium wallum. The percentage composition of 1,8-cineole is locationally independent (9 ± 1)%. The percentage composition of α-pinene and caryophyllene Z are locationally dependent, (20 ± 8)% and (8 ± 5)%, respectively. Figure 3 shows the locational variation of the percentage composition of the two major constituents of L. whitei: α-pinene is locationally independent (51 ± 7)% and E-ocimene is locationally dependent (9 ± 3)%. Green foliage was collected twice from 17 trees: once in spring 2010 and again in summer 2011. The mean difference in percentage oil yield (−0.0391%) was not significantly different in spring and summer for the same tree (paired t = −1.148; two-tailed *P* = 0.268; df = 16). However, the seasonal variation of the percentage oil yield from these 17 trees displayed in Figure 4, shows two trends: trees #7 and #16 (L. liversidgei chemotype I) have a greater percentage oil yield in spring and other Leptospermum trees have a greater percentage oil yield in summer. Statistical analysis of these hypotheses revealed: the mean difference in percentage oil yield (0.2375%) was significantly greater in spring for L. liversidgei chemotype I trees (paired t = 8.962; one-tailed *P* = 0.036; df = 1) and the mean difference in percentage oil yield (0.0759%) was significantly greater in summer for other Leptospermum trees (paired t = 2.940; one-tailed *P* = 0.006; df = 14). The mean difference in α-pinene percentage composition (2.7%) was significantly different in spring and summer for the same tree (paired t = 2.216; two-tailed *P* = 0.042; df = 16). Figure 5 shows the seasonal variation in the α-pinene percentage composition, but unfortunately does not reveal a direction for this difference.

In Additional file 1: Table S1 the range of percentage composition of compounds found and their retention indices are given for the Leptospermum species investigated in this work. Two principal components of L. juniperinum observed in this study (α-pinene, 21% and 1,8-cineole, 12–20%) were consistent with prior work [1]. Like previous studies [3], two extremes in chemical composition were identified in the L. liversidgei species in this work: chemotype I high in isopulegol (15–33%) and citronellal (19–44%); chemotype II high in neral (21%) and geranial (31%). The monoterpenoid nature of L. polygalifolium wallum observed in this study (α-pinene, 5–32%, β-pinene, 0–7% and 1,8-cineole, 4–13%) was consistent with prior work [4], as was their low concentration of sesquiterpene alcohols (α-eudesmol, 0–3%, β-eudesmol, 0–5% and γ-eudesmol, 0–3%). Like previous studies [2], the predominance of monoterpenes (α-pinene, 26–43%, β-pinene, 1–7%) in L. semibaccatum chemotype II and its significant amounts of globulol, 2–3%, spathulenol, 3–4%, α-cadinol, 3–7% and α-muurolol, 0.7–2%, were observed in this work. The principal component (α-pinene, 62%) of L. speciosum observed in this study was consistent with prior work [5]. L. trinervium chemotype I showed significant quantities of α-pinene, 40–67%, germacrene D, 0.8–2%, globulol, 1–3% and spathulenol, 4–6% in this work and these findings were consistent with literature values [2]. Like in previous studies [5], the main monoterpene hydrocarbon found in L. whitei was α-pinene, 42–63%. Five differences in geometric isomers have been identified between this work and previous studies. The first three differences are intra species specific, whilst the last two differences are for a particular constituent across the genus. In L. liversidgei chemotype I Brophy and co-workers [3] identified: isopulegol (8.6%) and an isopulegol (17.0%). In this work, percentage composition of isomers of isopulegol in L. liversidgei chemotype I have been confirmed as: isopulegol (neo) (10–22%), isopulegol (iso) (4–10%), isopulegol (neoiso) (0.6–1%) and isopulegol (8-hydroxy) (0–2%). Only farnesol (E,E) (4–37%) had previously been reported in L. laevigatum (5–39%) [2]. However, both farnesol (2Z,6Z) (0–0.4%) and farnesol (2Z,6E) (9–14%) have been identified in L. laevigatum in this work. Both farnesal (E,Z) (5.8%) and farnesal (E,E) (12.9%) were previously found in L. myrtifolium [1]. However, only farnesal (2E,6Z) (0.7–4%) was found in L. laevigatum in this study.

Caryophyllene E was discovered as a major constituent of L. juniperinum (5–31%) [1], L. laevigatum (5–39%) [2] and L. whitei (5–10%) [5]; as a minor constituent of L. liversidgei chemotype II (3.5%) [3], L. polygalifolium wallum (5–8%) [4], L. semibaccatum chemotype II (5–8%) [2] and L. trinervium chemotype I (7.6%) [2]; and as a trace constituent of L. liversidgei chemotype I (0.8%) [3]. In this work caryophyllene E was only identified as a trace constituent in L. polygalifolium wallum (0–0.1%), L. trinervium chemotype I (0–0.1%) and L. whitei (0–0.02%). Whereas caryophyllene Z was the isomer that predominated identification is this work: L. polygalifolium wallum (1–16%), L. semibaccatum chemotype II (3–13%), L. juniperinum (5–11%), L. trinervium chemotype I (0.6–7%), L. laevigatum (0.3–7%), L. whitei (0.7–5%), L. liversidgei chemotype I (0.3–4%), L. liversidgei chemotype II (3%) and L. speciosum (0.2%). The previous non-specific isomer identifications of humulene: L. juniperinum (0.5–10%) [1], L. laevigatum (0.3–3%) [2], L. trinervium chemotype I (1.5%) [2], L. whitei (0.5%) [5], L. liversidgei chemotype II (0.4%) [3], L. semibaccatum chemotype II (0.3%) [2], L. polygalifolium wallum (7–11%) [4] and L. speciosum (0.1%) [5]; are reflected in this work’s specific isomer identification of α-humulene: L. juniperinum (0.2–1%), L. laevigatum (0.1–0.4%), L. trinervium chemotype I (0.3–0.5%), L. whitei (0.1–0.5%), L. liversidgei chemotype II (0.3%), L. semibaccatum chemotype II (0.3–2%), L. polygalifolium wallum (0–1%) and L. speciosum (0.2%).

The genus wide prevalence of bicyclogermacrene observed in previous studies of Australian Leptospermum species was not seen in this study [1-5,15,16]. In fact bicyclogermacrene was not identified as a component in any Sunshine Coast and Northern Rivers Region Leptospermum species. The genus wide prevalence of α-himachalene and β-selinene observed in this study of Sunshine Coast and Northern Rivers Region Leptospermum species was not seen in prior work of Australian Leptospermum species and α-himachalene and β-selinene were observed in significantly smaller amounts in these previous studies [1-5,15,16]. α-pinene was observed in significantly higher proportions in the current work on L. laevigatum, L. liversidgei chemotypes I and II than in previous studies [2,3]. β-pinene was observed in significantly higher proportions in the current work on L. juniperinum and L. trinervium chemotype I than in previous studies [1,2].

Although significant amounts of δ-cadinene have been found in L. semibaccatum chemotype II previously [2], only traces of this compound were detected in the Sunshine Coast and Northern Rivers Region L. semibaccatum chemotype II investigated in this study. Similarly, significant amounts of p-cymene and p-cymen-8-ol have been found in L. trinervium chemotype I previously [2], but only traces of these components were identified in the Northern Rivers Region L. trinervium chemotype I examined in this work. In previous studies [5], spathulenol was the main oxygenated terpene and palustrol was a trace component found in L. whitei, but in this study palustrol was the main oxygenated terpene and spathulenol was the second most prevalent of these compounds in the Sunshine Coast and Northern Rivers Region L. whitei. Dauca-5,8-diene was contained in the Sunshine Coast and Northern Rivers Region L. semibaccatum chemotype II (5–12%) however this component was not present in previous studies of this chemotype [2]. In prior work [2], sabinene, 1,8-cineole, viridiflorene and viridiflorol were identified in significant quantities in L. trinervium chemotype I, but none of these components were detected in the Northern Rivers Region L. trinervium chemotype I examined in this work.

## Experimental

Green foliage from the number of individual trees listed in Additional file 2: Table S2 was sampled from each Leptospermum species. The locations, coordinates of the locations and collection season are also displayed in Additional file 2: Table S2. Leaf essential oils were obtained by steam distillation for 4 h. Hexane with an n-hexadecane internal standard was co-distilled with the samples and acted as a solvent trap for volatiles. GC-MS analyses were performed on a Varian 3900GC coupled to a Saturn 2100T mass spectrometer. The column was a Phenomenex ZB-5ms 30m × 0.25mm × 0.25mm. The carrier gas was constant flow 0.8mL/min He. The injection port was 260°C, with a split ratio of 100:1. The oven program operated at 50°C for 0.5 min, ramping at 3°C/min until 120°C, ramping at 1°C/min until 170°C, then ramping at 5°C/min until 220°C. Compound ionisation was at 70eV electron impact, analysing m/z + 30–300. Compounds were identified by a combination of their retention index, comparison of retention times to standards and comparison of their known mass spectra with published spectra [17].

## Conclusions

The percentage oil yield ranges and percentage composition of compounds for Leptospermum species found on the Sunshine Coast and Northern Rivers Region were for the most part commensurate with previous studies of these species. L. polygalifolium wallum showed significant locational variation in its percentage oil yield. L. liversidgei chemotype I showed higher percentage oil yield in spring which is in contrast to all other species investigated in this study which showed a percentage oil yield higher in summer. The percentage composition of α-pinene and caryophyllene Z in L. polygalifolium wallum samples and E-ocimene in L. whitei samples are locationally dependent. Brophy and co-workers’ [3] general identification of isopulegol in L. liversidgei chemotype I has been specified in this work as: isopulegol (neo) (10–22%), isopulegol (iso) (4–10%), isopulegol (neoiso) (0.6–1%) and isopulegol (8-hydroxy) (0–2%). Caryophyllene Z predominated identification in Leptospermum species found on the Sunshine Coast and Northern Rivers Region, but caryophyllene E predominated identification in previous studies of these species.

Although p-cymene can cause swelling of the bacterial cytoplasmic membrane and makes it more permeable for active compounds [8], L. trinervium chemotype I has too low an oil yield and only a trace amount of p-cymene, thus this action would be too negligible for phytomedical use. Similarly, although Garcinia brasiliensis with similar components to L. semibaccatum chemotype II showed anti-inflammatory activity [10], L. semibaccatum chemotype II has too low an oil yield for this activity to be sufficient for commercial phytomedical use. If the higher oil yielding and higher percentage composition of α-terpinene, γ-terpinene and 1,8-cineole plants of L. polygalifolium wallum could be cultivated independently, then the essential oils from these plants would have the potential to capitalise on the synergistic actions of: 1,8-cineole and terpinene to kill bacteria [9], and 1,8-cineole and morphine to reduce sensitivity to painful stimuli [10].

Essential oils high in neral and geranial have been found to inhibit the growth of methicillin-resistant Staphylococcus aureus [9] and the production of cytokines [12]. Neral and geranial vapours have been shown to provide antifungal activity against Phytophthora cactorum, Cryponectria parasitica and Fusarium circinatum [13]. Thus, L. liversidgei chemotype II, with its moderate oil yield and high concentrations of neral and geranial, has the potential for phytomedical use as an antibacterial, anti-inflammatory or antifungal agent.

Essential oils containing citronellal have been found to inhibit the growth of methicillin-resistant Staphylococcus aureus [9] and the production of nitric oxide [11]. 30% citronellal extract provided 78% repellency against mosquitoes in an in vitro test [14]. Thus, L. liversidgei chemotype I, with its high oil yield and high concentration of citronellal, has the potential for phytomedical use as an antibacterial or anti-inflammatory agent or insect repellent.

## Abbreviations

L = Leptospermum; SEQ = South East Queensland; NSW = New South Wales; RC = Respiratory Congestion.

## Competing interests

The authors declare that they have no competing interest.

## Authors’ contributions

SW collected green foliage samples, steam distilled green foliage samples into essential oils, analysed GCMS data and drafted the manuscript. PB collected green foliage samples and carried out GCMS of essential oils. Both authors read and approved the final manuscript.

## Author’s information

SW was awarded her PhD in Chemistry from the University of Queensland in 2010. Since that time she has worked as an Associate Lecturer of Science at the University of the Sunshine Coast. PB was awarded his PhD in Chemistry from the University of New South Wales in 1989. He then taught in the Department of Organic Chemistry, University of Adelaide for two years. From 1991 to 2000, Dr Brooks lectured analytical, general and organic chemistry at La Trobe University, Bendigo. Since that time he has worked as a Senior Lecturer in Chemistry at the University of the Sunshine Coast.

## Supplementary Material

Additional file 1: Table S1 Percentage composition of compounds found in Leptospermum essential oils. Click here for file

Additional file 2: Table S2 Location and collection season of Leptospermum green foliage samples. Click here for file
